# LncRNA NEAT1 remodels chromatin to promote the 5-Fu resistance by maintaining colorectal cancer stemness

**DOI:** 10.1038/s41419-020-03164-8

**Published:** 2020-11-09

**Authors:** Yihao Zhu, Hanqing Hu, Ziming Yuan, Qian Zhang, Huan Xiong, Zhiqiao Hu, Hongyu Wu, Rui Huang, Guiyu Wang, Qingchao Tang

**Affiliations:** Department of Colorectal Surgery, The Second Affiliated Hospital of Harbin Medical University, Harbin, China

**Keywords:** Cancer stem cells, Oncogenes, Cancer stem cells, Oncogenes

## Abstract

Resistance of chemotherapy is one of causes of recurrence and poor prognosis in patients with colorectal cancer (CRC). The role of differentially expressed long non-coding RNA (lncRNA) in 5-fluorouracil (5-Fu) resistance has not been fully elucidated. Here we observed that lncRNA NEAT1 was associated with 5-Fu resistance in CRC. Our Functional studies showed that NEAT1 promoted 5-Fu resistance in colorectal cells. In addition, A-TAC sequencing and chromatin immunoprecipitation (ChIP) showed that NEAT1 affected chromatin remodeling, increased the acetylation levels of histones, increased their enrichment at the promoters of ALDH1 and c-Myc, and promoted the expression of ALDH1 and c-Myc. Taken together, our study suggested that NEAT1 promoted 5-Fu resistance and cancer stemness by remodeling chromatin. Our finding provides a novel role of NEAT1 and may provide a new strategy for the treatment of CRC 5-Fu resistance.

## Introduction

Colorectal cancer (CRC) is the third leading cause of all-cancer-related death^1^. The treatment of CRC is mainly through surgery and drugs. 5-fluorouracil (5-Fu) is an effective drug for colorectal cancer^2^. So studying the mechanism of 5-Fu resistance may be a key factor in improving colorectal cancer survival^3^.

At present, more and more studies showed that cancer stem cells have the ability of self-renewal and partial differentiation^4–7^. They have some mechanisms to escape from damages caused by drugs, such as high drug transporter expression, efficient DNA repair, quiescence, and apoptotic block^8^. Thus, cancer stem cells are regarded as a main contributor of chemoresistance^9–12^.

LncRNAs are defined as RNA polymerase II transcripts longer than 200 nucleotides in length with limited coding potential^13,14^. NEAT1 is an essential component of nuclear paraspeckles and can participate in transcriptional regulation^15^. Studies had shown that lncRNA NEAT1 acted as a scaffold and recruited the chromosome modification enzyme EZH2 to silence target-specific genes, thereby promoting β-catenin nuclear transport and promoting the occurrence of gliomas^16^. In triple-negative breast cancer, lncRNA NEAT1 confered the oncogenic role through modulating chemoresistance and cancer stemness^17^. However, the biological role of NEAT1 on CRC cell 5-Fu chemoresistance remains poorly understood. In present study, we want to investigate the role of NEAT1 in CRC chemoresistance and reveal the way NEAT1 affect the resistance to 5-Fu.

## Materials and methods

All the human CRC tissues and paratumor normal tissues were collected in the Department of Colorectal Cancer Surgery, the Second Affiliated Hospital of Harbin Medical University. After surgical debulking, patients have undergone XELOX or mFOLFOX6 regimen therapy. Informed consent was obtained from the patients before sample collection in accordance with institutional guidelines. Recurrence was monitored by imaging examination systems (Chest X-ray and CT), gastrointestinal endoscopy with biopsy, and telephone follow-up. In order to obtain two independent cohorts for mutual verification, cohort A was collected from January 2012 to September 2012 and cohort B was collected from January 2014 to September 2014. The expression of NEAT1 was evaluated in cohort A and cohort B. The prognostic significance of NEAT1 was evaluated in cohort B. Patients were pathologically and clinically diagnosed with colorectal cancer. This study was carried out under the permission of the Clinical Research Ethics Committees of the Second Affiliated Hospital of Harbin Medical University.

### Cell lines and culture conditions

Human CRC cell lines HCT8 and HCT8-5FU were purchased from Shanghai Meixuan Corporation (Shanghai, China) and were cultured according to a previous report^18^. Human CRC cell line Lovo were purchased from Shanghai Institutes for Biological Sciences (Shanghai, China). Human CRC cell lines (HCT116, HT29, SW480, and SW620) and a normal colon epithelium cell line (NCM460) were purchased from the American Type Culture Collection (ATCC, Manassas, VA, USA). HCT116 and SW480 were grown in McCOY’s5A medium (Jiangsu Kaiji Biotechnology Co, Ltd) or Dulbecco’s modified Eagle’s medium (DMEM) medium (Gibco Laboratories, Grand Island, NY). All cells were cultured at 37 °C in a humidified incubator containing 5% CO_2_, supplemented with 10% fetal bovine serum (GIBCO, Carlsbad, CA).

### Cell transfection

Lenti-shNEAT1 and their corresponding control vectors were constructed and purchased from GeneChem (Shanghai, China). Following transfection, single-cell clonal isolates were selected in the presence of puromycin (Sigma-Aldrich Corp, St. Louis, MO, USA) for 2 weeks. The shRNA sequences are shown in Supplemental Table 3.

### CCK-8 assay

The cells were cultured in 96-well microporous plates (Corning, Life Sciences, USA) for 24, 48, and 72 h with 5-Fu at 10 μg/ml. The CCK-8 solution (Dojindo) was added to the cells and incubated at 37 °C for 2 h. The optical density (OD) was measured at 450 nm.

### RNA extraction and quantitative PCR

Total RNA was extracted from CRC cell lines with TRIzol reagent (Invitrogen, Carlsbad, CA, USA) and 2 μg total RNA was reverse-transcribed using the High Capacity cDNA Reverse Transcription Kit (Applied Biosystems, Foster City, CA) according to the manufacturer’s instructions. DNA was quantified using Nanodrop 2000 spectrophotometer (Thermo Fisher Scientific, Waltham, MA). Quantitative PCR (qPCR) and then 0.8 μg cDNA was applied for qPCR with SYBR Green reagent (Thermo Fisher Scientific, Waltham, MA) using the Applied Biosystem 7500 qPCR system (Applied Biosystems). Relative expression was compared by 2^−ΔΔCt^ method and β-actin served as the endogenous gene. Primers sequences were listed in Supplemental Table 4.

### Sphere formation

Cells (2 × 10^3^) were seeded into ultra-low attachment 24-well plates (Corning, Life Sciences, USA) and contained 1 × B27 (Gibco), 10 μg/l b-FGF (PeproTech), EGF 20 μg/l (PeproTech) in DMEM/F12 medium (GIBCO). All cells were cultured at 37 °C for 5 days in a humidified incubator containing 5% CO_2_ and 95% air. The pictures were taken by Nikon microscope and the diameters were calculated by ImageJ software.

### Flow cytometry and ALDEFLUOR assay

1 × 10^6^ cells were harvested and washed in PBS three times. Then, the cells were resuspended in 100 μl Flow Cytometry Staining Buffer (eBioscience). Two microliter of CD133 (STEM CELL Technologies Inc, Canada) were added and incubated for 20 min in dark. Next, the cells were washed by Flow Cytometry Staining Buffer and prepare to be detected. The CD133-positive subpopulation was detected by FACS (BD Accuri C5, USA).

For the ALDH assay, cells were identified using the ALDEFLUOR reagent kit (STEMCELL). Cells were suspended in ALDEFLUOR assay buffer at a concentration of 1 × 10^5^ cells/ml and divided into two tubes labeled “control” and “test”. Diethylaminobenzaldehyde (DEAB), a specific ALDH inhibitor, was added to the control tube to control for background fluorescence. Activated ALDEFLUOR reagent was added to tubes. The tubes were incubated for 30 min at 37 °C, and the tubes were then centrifuged for 5 min at 250 × *g*. Cell pellets were resuspended in ALDEFLUOR assay buffer and stored on ice. The ALDH1-positive subpopulation was detected by FACS (BD Accuri C5, USA).

### Western blotting assay and antibodies

Western blotting Proteins were separated on a 10% sodium dodecyl sulfate-polyacrylamide gel electrophoresis (SDS-PAGE) and transferred onto a nitrocellulose membrane. The blots were incubated with antibody overnight at 4 °C. Following three washes, membranes were then incubated with secondary antibody 2 h in room temperature. Signals were visualized by ECL (Beyotime, China).

The following antibodies were used: NANOG (Cell Signaling Technology #4903S), SOX2 (Cell Signaling Technology #3579S), c-Myc (Abcam #ab32072), OCT4 (Abcam #ab19857), H3K9me3 (Cell Signaling Technology #9751), H3K27me3 (Cell Signaling Technology #9733), H3K9ac (Cell Signaling Technology #9649), H3K27ac (Cell Signaling Technology #8173), GAPDH (ZSGB-Bio #TA-8), and Tubulin (ZSGB-Bio #TA-10).

### Chromatin immunoprecipitation (CHIP) assays

CHIP assays were performed using a Pierce Magnetic ChIP Kit (Thermo scientific #26157) according to the manufacturer’s instructions. Briefly, 2–4 × 10^6^ cells were crosslinked using 1% formaldehyde for 10 min at 25 °C and then sonicated in lysis buffer. After centriFugation, 10 μl of the supernatant was used as input; the remaining lysate was subjected to a ChIP assay using antibodies specific for H3K27ac (Cell Signaling Technology #8173).

### Immunohistochemistry (IHC) and scoring

IHC for target molecules was performed on serial sections from tumor tissues of CRC patients in cohort B. Tissue sections were deparaffinized, subjected to antigen retrieval using target antigen retrieval solution (Cell Signaling Technology #14747), and incubated with primary antibodies against ALDH1 (Cell Signaling Technology #54135; 1:200) and c-Myc (Abcam #ab32072; 1:200). Then sections were incubated with HRP-labeled antirabbit secondary antibodies (Thermo Fisher Scientific #31460). The immunostaining was observed and scored by two independent experienced pathologists.

The IHC staining was quantified as the *H*-score, which has been validated for many types of cancer^19–21^. The images were acquired using a imaging system (Pannoramic MIDI) under identical conditions. Scoring was performed using inForm software (Quant center). Typical images corresponding to negative (four images, scored as “0”), weak (four images, scored as “1”), intermediate (four images, scored as “2”), and strong (four images, scored as “3”) brown staining were selected by two independent experienced pathologist for software training. During training, an algorithm, which helps the software to distinguish between the cancer and noncancer tissues, and distinguish between the nuclear and cytoplasmic regions, and sets the thresholds for 0–3 staining, was optimized. Next, the optimized algorithm was used to perform scoring of the other samples. The *H*-score (between 0 and 300) for each sample was calculated in the following way: (% of cells stained at intensity 1 × 1) + (% of cells stained at intensity 2 × 2) + (% of cells stained at intensity 3 × 3). For ALDH1, 136 was the median level of the final scores of all cases. For c-Myc, 94 was the median level of the final scores of all cases. Stained tissues with a final score < median level was further classi fied as low, whereas tissues with a final score ≥ median level were determined as high.

### Statistical analysis

All statistical analyses were performed using SPSS 23.0 software (IBM). Data are expressed as the mean ± SD for at least three separate experiments. Unpaired two-tailed Student’s *t*-test between two groups and one-way ANOVA followed by Tukey’s post hoc test between multiple groups were applied. Prism software version 6 (GraphPad software) was used to plot. Differences were considered as significant where *P* < 0.05 represented as *, *P* < 0.01 represented as **, and *P* < 0.001 represented as ***.

## Results

### NEAT1 is associated with colorectal cancer recurrence and patient outcome

**Fig. 1:**
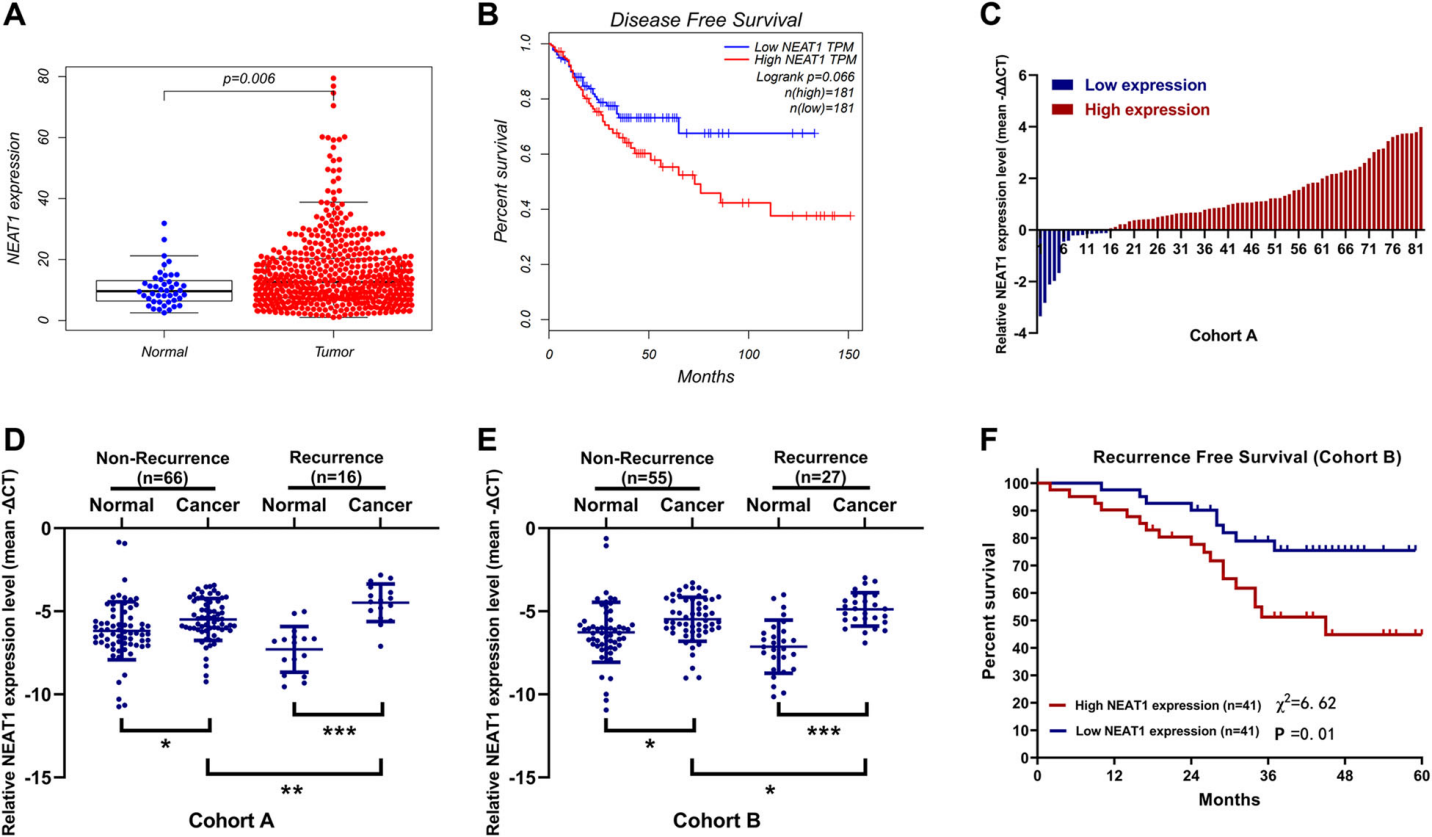
NEAT1 expression in colorectal cancer patients. **A** Analysis of expression patterns of NEAT1 in cancer and normal tissues from TCGA dataset. **B** Kaplan–Meier analysis of DFS of CRC patients from TCGA dataset. **C**–**E** Relative expression of NEAT1 in primary and recurrent CRC tissues. The NEAT1 expression was normalized to β-actin. *P < 0.05, **P < 0.01, and ***P < 0.001. **F** Kaplan–Meier analyses of the associations between NEAT1 expression level and recurrence free survival (RFS) of patients with CRC (the log-rank test was used to calculate *P*-values).

To study the role of NEAT1 in CRC, we first detected its expression in CRC tissues and normal tissues in The Cancer Genome Atlas (TCGA) datasets. TCGA shown elevated NEAT1 levels in human CRC tissue relatived to normal tissue (Fig. 1A). Next, Kaplan–Meier analysis was used to determine whether NEAT1 expression levels in the CRC tissues were associated with clinical patient outcome. Survival analysis of the TCGA cohort revealed that a higher NEAT1 level was associated with poor disease-free survival (DFS) in CRC patients (Fig. 1B). Next, we measured NEAT1 levels in 66 pairs of normal tissues and tumor tissues of CRC patients without recurrence, and 16 pairs of normal tissues and tumor tissues of CRC patients with recurrence by qRT-PCR. As shown in Fig. 1C, the expression level of NEAT1 in tumor tissues of most patients is significantly higher than that of normal tissues. And we can see that the expression level of NEAT1 in tumor tissues of patients with recurrence is higher than that of patients without recurrence (Fig. 1D), whose clinical characteristics were shown in Supplemental Table 1. Next, we used another cohort B (55 pairs of normal tissues and tumor tissues of CRC patients without recurrence, and 27 pairs of normal tissues and tumor tissues of CRC patients with recurrence) of our center to validate the results of cohort A, and the results were consistent (Supplemental Table 2 and Fig. 1E). Then we evaluated the prognostic significance of NEAT1 in cohort B. Patients in the NEAT1-high-expression group showed a shorter recurrent free survival (RFS) than those in the NEAT1-low-expression group (*P* = 0.01, Fig. 1F). This suggests that NEAT1 may play a role in CRC recurrence.

### NEAT1 promotes 5-Fu resistance in colorectal cancer cells

**Fig. 2:**
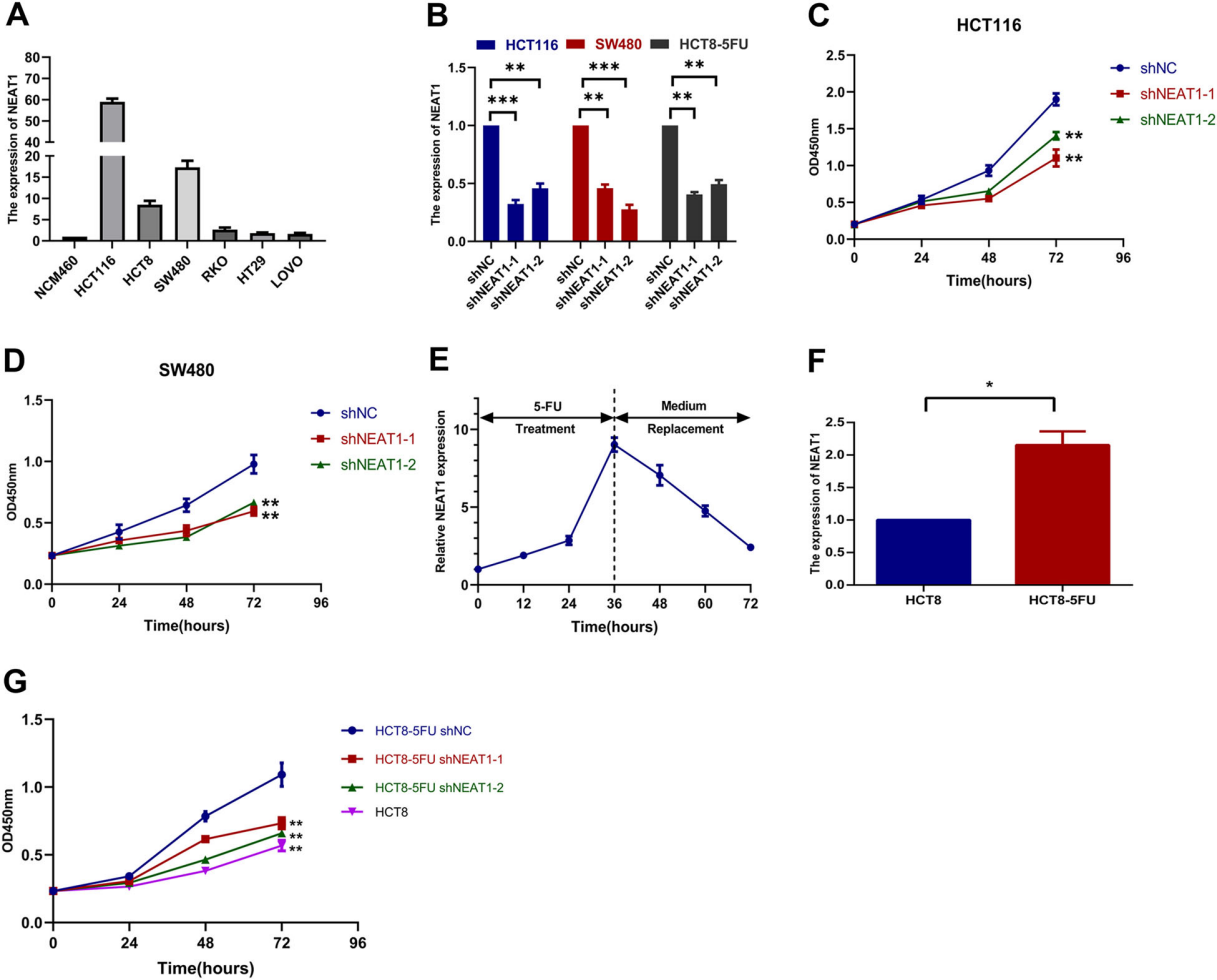
NEAT1 promotes 5-Fu resistance in CRC cells. **A** Relative expression of NEAT1 in CRC cell lines and the normal colon epithelium cell line NCM460 by qRT-PCR. Data are presented as mean ± SD from three independent experiments. **B** Validation of knockdown efficacy of NEAT1 in CRC cell line by qRT-PCR. ***P < 0.001. **C**, **D** CCK8 assays showing that knockdown of NEAT1 increased sensitivity to 5-FU in the HCT116 and SW480 cell lines. *P < 0.05, **P < 0.01, and ***P < 0.001. **E** Dynamic changes of NEAT1 expression level in response to 5-Fu treatment (10 μg/ml). The 1–6 times courses successively present 12, 24, and 36 h after 5-Fu treatment and 48, 60, and 72 h after medium replacement. **F** Relative expression of NEAT1 in 5-Fu resistant HCT8-5Fu cell line and normal HCT8 cell line by qRT-PCR. *P < 0.05. **G** CCK8 assays showing that knockdown of NEAT1 increased sensitivity to 5-FU in the HCT8-5FU cell lines (*P < 0.05, **P < 0.01, and ***P < 0.001).

We measured the expression of NEAT1 in each cell line and found that it was higher in HCT116 and SW480 than other cell lines (Fig. 2A). Subsequently, we constructed NEAT1 knockdown stable cell lines for three cell lines, HCT116, SW480, and HCT8-5FU (Fig. 2B). With 10 μg/ml of 5-Fu treatment, CCK-8 assays showed that knockdown of NEAT1 could decrease the resistance of colorectal cancer cell lines to 5-Fu (Fig. 2C, D). In addition, in the drug-sensitive cell line HT29, NEAT1 expression increased significantly and reached its peak at 36 h after drug treatment, and gradually decreased after the replacement of normal medium (Fig. 2E), indicating that NEAT1 was involved in the response to 5-Fu treatment. We performed experiments on 5-Fu resistance cell lines (HCT8-5FU). QRT-PCR showed that the expression of NEAT1 in HCT8-5FU cells was higher than that in normal HCT8 cell line (Fig. 2F). Moreover, we found that the resistance of HCT8-5FU cells to 5-Fu also decreased after knockdown of NEAT1 (Fig. 2G).

### NEAT1 affectes stemness maintenance of colorectal cancer cells

**Fig. 3:**
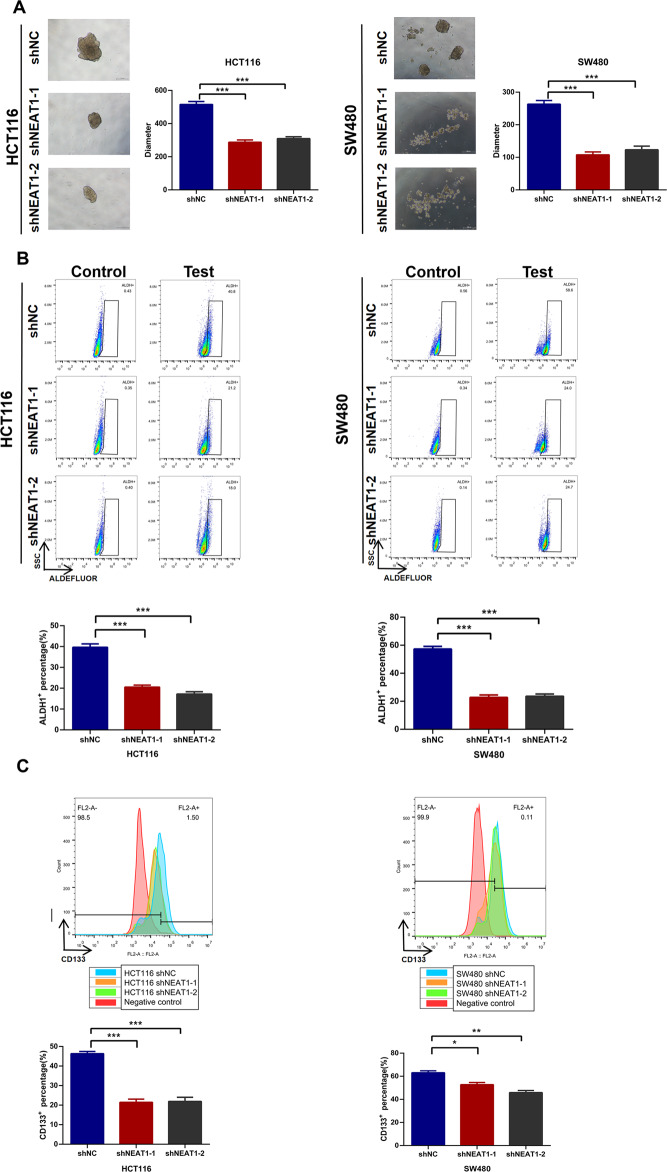
Downregulation of NEAT1 attenuates CSC properties in colorectal cancer cells. **A** HCT116 and SW480 cells when treated with shRNA NEAT1 were plated in low-serum, non-adherent culture conditions.Images were obtained after 7 days in culture. Sphere size are shown (***P < 0.001). **B** The ratio of ALDH1-positive cells in HCT116 and SW480 cells when treated with shRNA NEAT1 are shown. The data are expressed as the mean ± S.D. of three independent experiments (**P < 0.01, ***p < 0.001). **C** The ratio of CD133-positive cells in HCT116 and SW480 cells when treated with shRNA NEAT1 are shown. The data are expressed as the mean ± S.D. of three independent experiments (**P < 0.01, ***P < 0.001).

Cancer stem cells are main cause of chemoresistance. Then we explored whether NEAT1 had an effect on CRC stemness. Knocking down NEAT1 significantly reduced its spheroid diameter (Fig. 3A). Next, using the ALDEFLUOR assay, we found that knockdown of NEAT1 decreased activity of ALDH1 in CRC cells (Fig. 3B). Moreover, knockdown of NEAT1 could also suppressed CD133 expression on cell surface (Fig. 3C).

### Knockdown of NEAT1 leads to widespread changes in chromatin accessibility

**Fig. 4:**
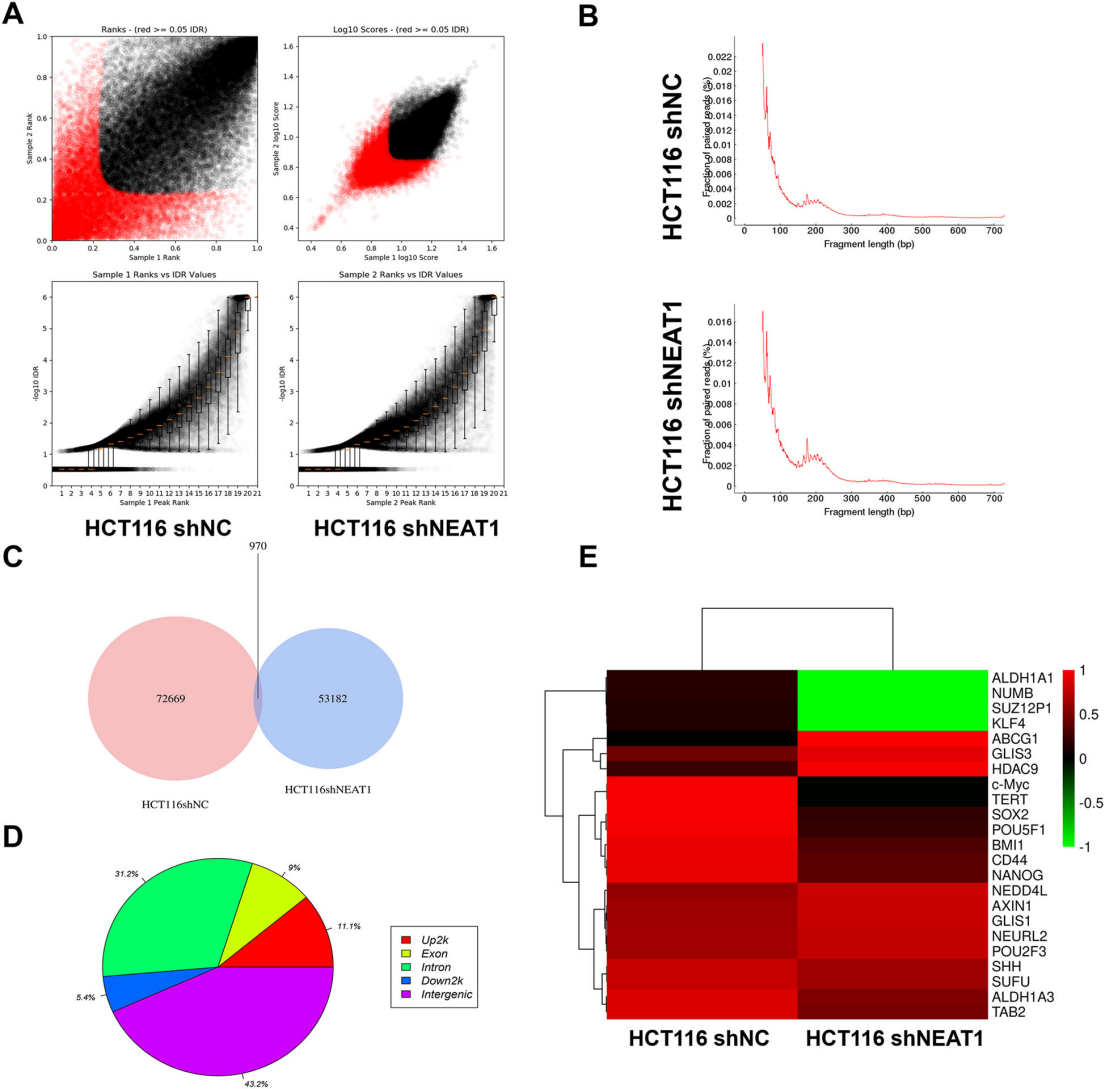
ATAC-Seq showed that knockdown of NEAT1 may affect the chromatin remodeling of colorectal cancer cells. **A** The irreproducible discovery rate (IDR) analysis of the sample. **B** DNA fragment length distribution measured in two samples. **C** Peak comparison between the two groups. **D** Distribution of different peaks across genomic features. **E** Hierarchical clustering of normalized counts of differentially expressed genes.

In recent years, ATAC-seq has been used more and more in basic research as a method for determining genome-wide chromatin accessibility^22,23^. First, we performed the irreproducible discovery rate (IDR) analysis on the peaks detected by each repeated sample sent to evaluate the repeatability of the experiment and the accuracy of the peaks. We found that the quality evaluation of the samples was qualified (Fig. 4A). According to the length distribution of DNA fragments, there was no significant difference between them (Fig. 4B). We then analyzed the peaks and found that 970 of them were the same (Fig. 4C). We distributed the different peaks between the two groups on the genome (Fig. 4D). Then we selected some genes related to stemness in different peaks. We could see that the genes were significantly reduced after NEAT1 was knocked down (Fig. 4E). In summary, we speculated that the knockdown of NEAT1 may affect histone modifications or transcription factor enrichment at the gene transcription start site and thus affect the stemness of CRC cells.

### NEAT 1 affects the expression of stem-related genes in colorectal cancer cells

**Fig. 5:**
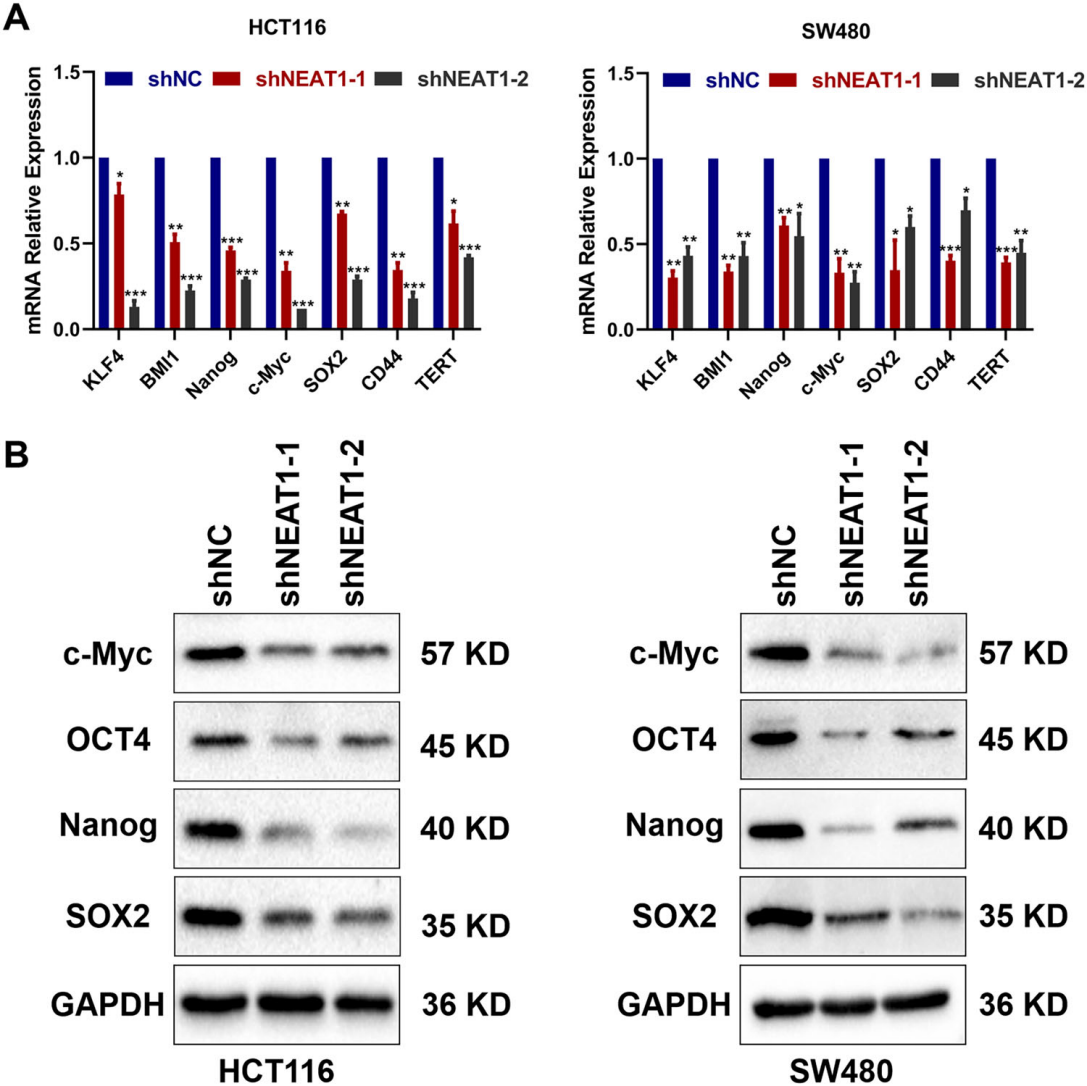
NEAT1 affected the CSC propertiesof colorectal cancer cells by affecting the expression of stemness-related genes. **A** Relative expression of genes in HCT116 and SW480 cells transfected with shRNA NEAT1 was determined by qRT-PCR. **B** Detection of relative expression levels of proteins in HCT116 and SW480 cells transfected with shRNA NEAT1 by Western blotting.

Based on the ATAC-seq, we speculated that NEAT1 may affect the stemness of CRC by affecting the expression of stemness-related genes. QRT-PCR indicated that downregulation of NEAT1 inhibited mRNA levels of these stemness factors (Fig. 5A). Western blotting assay also showed that SOX2, NANOG, c-Myc, and OCT4 were also decreased after the downregulation of NEAT1 (Fig. 5B). In summary, these data indicated that knockdown of NEAT1 may decrease the expression of stemness factors to suppress CSC properties of colorectal cancer cells.

### NEAT1 leads to acetylation of H3K27 in the promoter region of ALDH1 and c-Myc in colorectal cancer and is correlated with patient response to 5-FU treatment

**Fig. 6:**
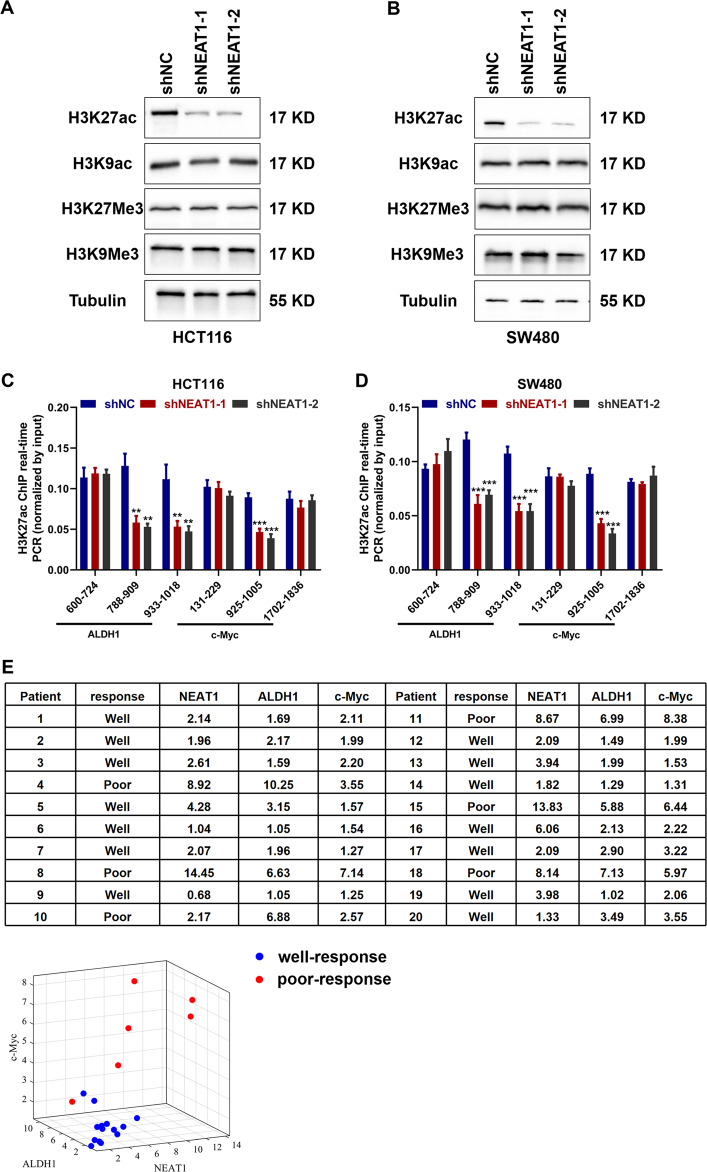
NEAT1 affects the level of acetylation in the promoter region of gene and is correlated with patient response to 5-Fu treatment. **A**, **B** Distribution of reads near the transcription start site of ALDH1 and c-Myc. **C**, **D** ChIP-qPCR showed H3K27ac occupancy levels in the ALDH1 and c-Myc promoter regions in different groups. **E** The expression of NEAT1, ALDH1 and c-Myc of 20 patients who receive XELOX as neoadjuvant chemotherapy (Relative expression was compared by 2^−ΔΔCt^ method and β-actin served as the endogenous gene, the values in the table were taken as −2^−ΔΔCt^.).

Studies have shown that NEAT1 can be used as a scaffold to participate in the chromatin remodeling of glioma cells, promote the increase of the trimethylation level of the promoter of downstream genes, and then promote its expression^16^. LncRNA NEAT1 is a component of nuclear paraspeckle, so we therefore speculated whether NEAT1 could also affect chromatin remodeling and histone modification levels of colorectal cancer cells. By extracting nuclear proteins from stably transfected cells, we found that the level of H3K27ac after NEAT1 knockdown was significantly lower than that of the control group, which implied the H3K27ac may influence the transcription of ALDH1 and c-Myc (Fig. 6A, B). To reveal how NEAT1 regulated the expression of ALDH1 and c-Myc through histone modification, we performed ChIP-qPCR to detect their regulation mechanism. The results showed that knockdown of NEAT1 group decreased the H3K27ac binding ability in HCT116 cell line (Fig. 6C) and SW480 cell line (Fig. 6D). These results indicate that NEAT1 led to acetylation of H3K27 in the promoter region of ALDH1 and c-Myc in colorectal cancer. In order to investigate the correlation of NEAT1, ALDH, and c-MYC expression with patient response to 5-FU treatment, we selected 20 patients who receive XELOX as neoadjuvant chemotherapy. According to the RECIST criteria, the complete response and partial response was regarded as well-response and stable disease and progressive disease were regarded as poor-response. Then, we performed PCR assay to evaluate the expression of NEAT1, ALDH1, and c-Myc. The results showed that patients with lower expression have better efficacy, and patients with higher expression have worse effects on 5-Fu treatment (Fig. 6E).

### ALDH1 or c-Myc and NEAT1 co-expression have comparatively poorer prognoses

**Fig. 7:**
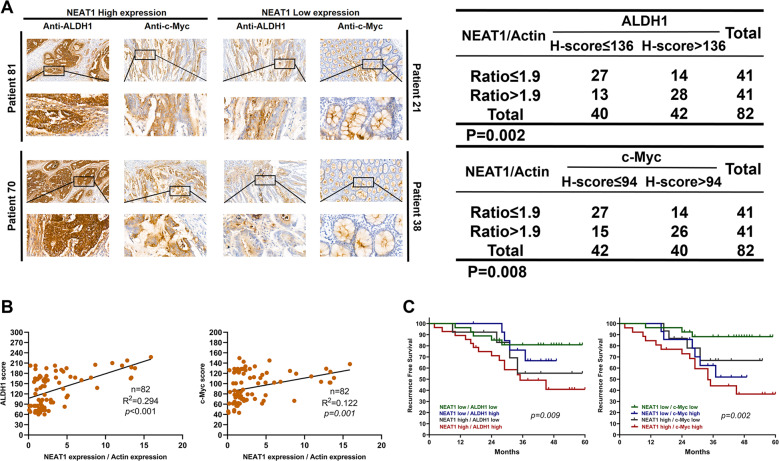
The correlation between ALDH1 expression, c-Myc expression, and NEAT1 expression in CRC tissues. **A** Representative IHC staining of ALDH1 or c-Myc and NEAT1 in serial sections are shown. **B** NEAT1 overexpression was significantly positively correlated with ALDH1 and c-Myc upregulation in human CRC tissues. Spearman correlation analysis was used. **C** Combination of ALDH1 or c-Myc and NEAT1 had a higher probability of poor prognosis than either factor alone. *P* is based on the log-rank test.

To further examine the relationship between c-Myc or ALDH1 expression and NEAT1 in clinical samples, we performed immunohistochemistry (IHC) staining to measure c-Myc and ALDH1 in tissue specimens of cohort B. ALDH1 and c-Myc exhibited higher expression in NEAT1 high expression tumor tissue specimens compared to the low NEAT1 expression tissues (Fig. 7A). Then we investigated the association between ALDH1 or c-Myc and NEAT1 expression in CRC. ALDH1 or c-Myc and NEAT1 expression were positively correlated in CRC tissues (Fig. 7B). Moreover, patients presenting with ALDH1 or c-Myc and NEAT1 co-expression had comparatively poorer prognoses (Fig. 7C).

## Discussion

Colorectal cancer is the leading cause of cancer deaths worldwide, and 5-Fu based chemotherapy has been widely used to treat different types of cancer including CRC. Understanding the mechanisms of resistance in CRC is imperative to improve the survival. In recent years, studies have shown that NEAT1 was associated with resistance to chemotherapy in hepatocellular carcinoma^24^, endometrial cancer^25^ and others. Moreover, the effects of NEAT1 and cancer stem cells in breast cancer have also been reported^17^. In this study, we observed that NEAT1 was highly expressed in colorectal cancer tissues from patients with recurrence and was also associated with poor recurrence-free survival. Thus, NEAT1 may be related to patients’ drug resistance and recurrence.

To elucidate the underlying mechanism, further research revealed that downregulation of NEAT1 significantly inhibited the growth of CRC cell lines and reduced the sensitivity to 5-Fu. At present, more and more studies have shown that cancer stem cells can evade the damage of chemotherapy drugs through some mechanisms^8^. We speculated that NEAT1 may mediate drug resistance by regulating the stemness of CRC. Further research showed that the activity of ALDH1 and CD133 was decreased by downregulating the expression of NEAT1 in colorectal cancer cell lines. Next, we found that downregulation of NEAT1 decreased the expression of stemness factors, such as SOX2, NANOG, c-Myc, and OCT4. In summary, knockdown of NEAT1 could reduce the expression of stemness factors to inhibit CSC properties of colorectal cancer cells.

NEAT1 is a component of nuclear paraspeckle, so we speculated that knockdown of NEAT1 may affect chromatin remodeling. Studies have shown that NEAT1 can be used as a scaffold to participate in the chromatin remodeling of glioma cells, promote the increase of the trimethylation level of the promoter of downstream genes, and then promote its expression^16^. In our study, we found that NEAT1 increased H3K27ac by affecting chromatin remodeling and led to an increase in acetylation levels of ALDH1 and c-Myc promoter regions, which increased their expression and thus enhanced the stemness of colorectal cancer cells.

In summary, our work demonstrated that NEAT1 was associated with 5-Fu resistance in CRC patients, suggesting that NEAT1 may affect 5-Fu resistance in colon cancer cells by affecting cancer cell stem. In addition to its biological importance, our work may be related to the clinical management of CRC patients. Our data raise an important clinical question: Are conventional chemotherapy regimens, including 5-Fu, suitable for CRC patients with high NEAT1 expression? Alternatively, we suggest that traditional chemotherapy be combined with drugs that target tumor stem cells to treat CRC patients with high levels of NEAT1.

## Supplementary information

Supplemental Table
