# Supplementary material for: LncRNA NEAT1 remodels chromatin to promote the 5-Fu resistance by maintaining colorectal cancer stemness
Source: Cell Death Dis. 2020 Nov 9;11(11):962. doi: 10.1038/s41419-020-03164-8 (PMC7653927; doi:10.1038/s41419-020-03164-8)
Supplement: Supplementary file 1 — Supplemental Table [file 41419_2020_3164_MOESM1_ESM.doc]

**Supplemental Table 1 Clinicopathological characteristics of colorectal cancer patients in cohort A**

TNM according to staging TNM of American Joint Committee on Cancer (AJCC) in 2010

| Characteristics | Total | Non-Recurrence(n=66) | Recurrence  (n=16) | *P value* |
| --- | --- | --- | --- | --- |
| Gender |  |  |  | 0.182 |
| Female | 43 | 37 (56.1%) | 6 (37.5%) |  |
| Male | 39 | 29 (43.9%) | 10 (62.5%) |  |
| Age (year) |  |  |  | 0.817 |
| ≥55 | 44 | 35 (53.0%) | 9 (56.2%) |  |
| <55 | 38 | 31 (47.0%) | 7 (43.8%) |  |
| Tumor size (cm) |  |  |  | 0.836 |
| ≥5 | 34 | 27 (40.9%) | 7 (43.8%) |  |
| <5 | 48 | 39 (59.1%) | 9 (56.2%) |  |
| Differentiation |  |  |  | 0.033 |
| Moderate/poor | 53 | 39 (59.1%) | 14 (87.5) |  |
| Well | 29 | 27 (40.9%) | 2 (12.5%) |  |
| Depth of tumor |  |  |  | 0.334 |
| T1+T2 | 29 | 25 (37.9%) | 4 (25.0%) |  |
| T3+T4 | 53 | 41 (62.1%) | 12 (75.0) |  |
| Lymph node metastasis (N) |  |  |  | 0.565 |
| N0 | 36 | 30 (45.4%） | 6 (37.5%) |  |
| N1 or above | 46 | 36 (54.6%) | 10 (62.5%) |  |

**Supplemental Table 2 Clinicopathological characteristics of colorectal cancer patients in cohort B**

TNM according to staging TNM of American Joint Committee on Cancer (AJCC) in 2010

| Characteristics | Total | Non-Recurrence(n=55) | Recurrence  (n=27) | *P value* |
| --- | --- | --- | --- | --- |
| Gender |  |  |  | 0.697 |
| Female | 42 | 29 (52.7%) | 13 (48.1%) |  |
| Male | 40 | 26 (47.3%) | 14 (51.9%) |  |
| Age (year) |  |  |  | 0.796 |
| ≥55 | 50 | 33 (60.0%) | 17 (63.0%) |  |
| <55 | 32 | 22 (40.0%) | 10 (37.0%) |  |
| Tumor size (cm) |  |  |  | 0.386 |
| ≥5 | 43 | 27 (49.1%) | 16 (59.3%) |  |
| <5 | 39 | 28 (50.9%) | 11 (40.7%) |  |
| Differentiation |  |  |  | 0.005 |
| Moderate/poor | 56 | 32 (58.2%) | 24 (88.9%) |  |
| Well | 26 | 23 (41.8%) | 3 (11.1%) |  |
| Depth of tumor |  |  |  | 0.787 |
| T1+T2 | 29 | 20 (36.4%) | 9 (33.3%) |  |
| T3+T4 | 53 | 35 (63.6%) | 18 (66.7%) |  |
| Lymph node metastasis (N) |  |  |  | 0.380 |
| N0 | 39 | 26 (47.3%） | 10 (37.0%) |  |
| N1 or above | 43 | 29 (52.7%) | 17 (63.0%) |  |

**Supplemental Table 3. Sequences of shRNA**

| sgRNA | Sequence 5’---3’ |
| --- | --- |
| NEAT1 shRNA1 | CACCTGTTTGCCTGCCTTCTT |
| NEAT1 shRNA2 | ACGCAGCAGATCAGCATCCTT |
| scramble for shRNA | TTCTCCGAACGTGTCACGT |

**Supplemental Table 4. PCR primer sequences**

| Primer name | Sequence 5’---3’ |
| --- | --- |
| NEAT1 FW | TTTGTGCTTGGAACCTTGCT |
| NEAT1 RW | TCAACGCCCCAAGTTATTTC |
| KLF4 FW | CCCACATGAAGCGACTTCCC |
| KLF4 RW | CAGGTCCAGGAGATCGTTGAA |
| BMI1 FW | CGTGTATTGTTCGTTACCTGGA |
| BMI1 RW | TTCAGTAGTGGTCTGGTCTTGT |
| NANOG FW | AATGGTGTGACGCAGGGATG |
| NANOG RW | TGCACCAGGTCTGAGTGTTC |
| c-Myc FW | CGACGAGACCTTCATCAAAAAC |
| c-Myc RW | CTTCTCTGAGACGAGCTTGG |
| SOX2 FW | TGAACGGCTCGCCCACCTAC |
| SOX2 RW | AGCTGGCCTCGGACTTGACC |
| CD44 FW | CTGCCGCTTTGCAGGTGTA |
| CD44 RW | CATTGTGGGCAAGGTGCTATT |
| TERT FW | AAATGCGGCCCCTGTTTCT |
| TERT RW | CAGTGCGTCTTGAGGAGCA |
| c-Myc H3K27ac ChIP FW 131-229 | AGCCTGGGTGACAGAAGGAGAC |
| c-Myc H3K27ac ChIP RW 131-229 | GAGGCACAGCAGAAGGTGATGG |
| c-Myc H3K27ac ChIP FW 925-1005 | ACCCATCTTGAACAGCGTACATGC |
| c-Myc H3K27ac ChIP RW 925-1005 | TCTCCCTCCACCACCTCCAAAAG |
| c-Myc H3K27ac ChIP FW 1702-1836 | TGAGAAATTGGGAACTCCGTGTGG |
| c-Myc H3K27ac ChIP RW 1702-1836 | GCGTCTGTTTAGCCCTGAGATGTG |
| ALDH1 H3K27ac ChIP FW 600-724 | CAGATTGCACATCGGCAGCA |
| ALDH1 H3K27ac ChIP RW 600-724 | TGAGGAGGTGAGGGAGAGGA |
| ALDH1 H3K27ac ChIP FW 788-909 | CCTAGCACTTTGGGAGGCCA |
| ALDH1 H3K27ac ChIP RW 788-909 | GTGCCCCTATGCCTGGCTAA |
| ALDH1 H3K27ac ChIP FW 933-1018 | AGAGGCTGAGGCAGGAGGAT |
| ALDH1 H3K27ac ChIP RW 933-1018 | CACCCAGGCTAGAGTGCAGT |
